# Beyond AIC: An Interpretive Descriptive Qualitative Study of Youth Experiences and Perceptions of Living With Type 2 Diabetes

**DOI:** 10.1111/jan.70230

**Published:** 2025-09-25

**Authors:** Mandy M. Archibald, Allison Dart, Brandy Wicklow, Katherine J. Pundyk, Seth D. Marks, Elizabeth A. C. Sellers

**Affiliations:** ^1^ College of Nursing and Health Sciences University of Manitoba Winnipeg Manitoba Canada; ^2^ Children's Hospital Research Institute of Manitoba Winnipeg Manitoba Canada; ^3^ 99 Curry Place, Helen Glass Centre for Nursing, University of Manitoba Winnipeg Manitoba Canada; ^4^ Department of Pediatrics and Child Health University of Manitoba Winnipeg Manitoba Canada

## Abstract

**Objective:**

To generate an in‐depth understanding of the perceptions and experiences of individuals with youth‐onset type 2 diabetes (T2D) to inform knowledge translation initiatives and clinical care.

**Design:**

Interpretive descriptive qualitative study.

**Methods:**

Individuals were eligible to participate if they received a T2D diagnosis on or before 18 years of age, resided in Manitoba, and were between 10 and 25 years of age at the time of data collection. Twenty‐two individuals (13 females, 7 males, 2 prefer not to indicate gender; mean age = 19.3 years) participated in 22 semi‐structured interviews (mean length: 29:01 min) remotely using Zoom video conferencing software or by telephone. Data were analysed using inductive thematic analysis.

**Results:**

Four themes were generated: (1) Low public knowledge, misconceptions, and stigma impact youth experiences including those of diagnosis, disclosure, treatment, and supports; (2) shared familial experiences impacts perception of the future; (3) mental and emotional wellness is critically important but requires more attention; and (4) T2D carries unanticipated positive and negative impacts for youth.

**Conclusions:**

Findings illustrate the complex interrelationships between public and personal conceptions of T2D, stigma, and T2D navigation, emphasising the centrality of emotional and mental well‐being to participants' T2D experiences and management. This representation of experiences and perceptions of youth onset T2D offers direction for holistic and youth‐centred research and care and highlights areas where further mental health and educational resources would be beneficial.

**Patient and Public Contribution:**

The knowledge translation resource being developed from this study involves input from patient and public partners.

Type 2 diabetes (T2D) is no longer regarded as a disease of adulthood. With rising global prevalence, youth onset T2D (diagnosed < 18 years of age) is now considered a global public health emergency (Dabelea et al. 2014; Viner et al. 2017; Zeitler et al. 2018). Prevalence of youth onset T2D has been increasing across North America over the last few decades (Lynch et al. 2021), and in the central Canadian province of Manitoba, the incidence of youth onset T2D rose from 16/100,000 per year in 2009–2010 to 31.1/100,000 per year in 2017–2018, while prevalence increased from 66.4 to 124.2/100,000 during this same period (Sellers et al. 2024). Although published epidemiological data on youth onset T2D vary by country and are infrequently reported, Lynch et al. (2021) investigated prevalence data and found it has been reported at country‐specific levels for 12 countries, with additional publications documenting prevalence at a different level (e.g., single site; Lynch et al. 2021). These data demonstrate high prevalence of youth onset T2D in China and the United States of America, while Taiwan and the United Kingdom report high incidence (Lynch et al. 2021). Tackling this public health emergency requires sensitivity to patient‐centered issues—such as illness perceptions and self‐care practices—which have received little attention to date (Agarwal et al. 2018). This is especially critical as youth onset T2D is a heterogeneous and aggressive form of diabetes, marked by early and progressive β‐cell dysfunction that contributes to worsening glycemic control over time (Zeitler et al. 2018). With the elevated risk for early‐onset complications faced by youth with T2D (e.g., micro and macrovascular complications), understanding youth experiences and factors that may affect perceptions of wellness as well as self‐management is critical to advancing a patient‐centered research agenda and clinical care (Archibald et al. 2021; Dart et al. 2014).

Youth facing food insecurity, social disadvantage, and poverty represent a substantial proportion of youth living with T2D (McGavock et al. 2017), and First Nations youth are disproportionately affected (Sellers et al. 2024). Such contextual factors underscore the complexity of psychosocial perspectives and experiences of youth onset T2D, which have been shown to influence wellbeing and treatment outcomes (Agarwal et al. 2018; Nadeau et al. 2016). Despite this knowledge, robust qualitative studies in this domain are lacking, which limits understanding of youth‐centred issues and experiences necessary to advance holistic research and care. Here, we present the results of an interpretive qualitative study which explored youth perceptions and experiences of T2D, to identify support needs, barriers, and facilitators to self‐management.

## 1 Qualitative Investigations of Youth Onset T2D

Although quantitative research has dominated the research landscape of youth onset T2D, qualitative investigations have also emerged. Collectively, these studies attend to life transitions across geographic locations (e.g., Rasmussen et al. 2016), self‐management (Huynh 2018; Mulvaney et al. 2008), social support experiences (Brouwer et al. 2012; Huynh 2016), and generally investigate a smaller number of youth participants (Protudjer et al. 2014; Salamon et al. 2012; Wicklow et al. 2021). Additionally, a recent interview study with a mixed sample of 24 participants (including 8 adolescents) with T2D was conducted in Western Australia (Carman et al. 2023). Like the work of Huynh (2016, 2018), this work noted shame, judgement, and concealment as components of the youth experience, warranting further exploration within a robust Canadian sample.

Although the above studies provide valuable insights, they also underscore the need for further research in other contexts. Specifically, the work of Rasmussen et al. (2016) explored the psychosocial similarities and differences between Australian and Danish young adults, highlighting comparable impacts on life transitions between these two geographic groups. Huynh's (2018) grounded theory of 8 participants (7 females) suggested a dominance of physical care emphasis in care encounters and highlighted the relevance of mental health to T2D, warranting deeper investigation into these perceptions in a larger and more heterogeneous sample such as in the current study. Data collection in the study by Mulvaney et al. (2008) occurred over 20 years ago (2003, 2005, respectively) and while it contributed early understandings towards self‐management, it did not explore perceptions of T2D or experience of youth more generally. Conversely, Salamon et al. (2012) did explore perceptions of T2D in terms of its impact on daily life, adjustments, and self‐care; however, the sample was predominantly female (75%) and the limited sample of 8 participants suggests more investigation into T2D adjustments may be warranted. Further, the short interval between diagnosis and data collection fails to capitalise on the benefit of actual adjustment and retrospective reflection. The grounded theory by Protudjer et al. (2014) sought to triangulate perspectives of youth with T2D, their primary caregivers, and healthcare professionals to explore antecedents to adopting lifestyle approaches. Various themes such as supportive relationships and social determinants of health were seen to impact behavioural change. Overall, general perspectives and experiences surrounding youth onset T2D held by young people in North America are not well reflected in current research; robust and contemporary investigations are lacking. As such, we aimed to explore: (1) how youth with T2D understand and perceive their condition and the (2) nuanced experiences to identify encrypted information needs, barriers, and facilitators to self‐management. Our intention was for these findings to inform the collaborative design of a knowledge translation (KT) resource targeting knowledge and misconceptions around youth onset T2D.

## 2 Methods

### 2.1 Design

This is an exploratory qualitative study guided by Interpretive Description, which helps generate understandings of clinically relevant phenomena in relation to its context (Thorne 2008). Methods are summarised here and detailed in our published protocol (Archibald et al. 2021).

### 2.2 Theoretical Framework

The Canadian Institutes of Health Research Knowledge‐to‐Action‐Ethics framework (Canadian Institute of Health Research 2018) provided guidance for this study and reflects our attention to the ethical nuances of research with youth, overlaid by complexities of social stigma often experienced by youth living with T2D. The framework aligns with the concurrent knowledge generation and mobilisation aims of this project and encouraged critical reflection throughout the research process (e.g., whose voices are represented in this work; what historical and contextual factors are likely to influence inquiry and its outcomes) through planned dissemination (e.g., what influences the selection of evidence). This framework influenced the overall study design and encouraged critical questioning of the findings as we move towards the KT objectives.

### 2.3 Setting, Sample and Recruitment

Individuals were eligible to participate if they received a T2D diagnosis on or before 18 years of age, resided in Manitoba, and were between 10 and 25 years of age at the time of data collection. We included participants who were young adults at the time of interviewing to benefit from the retrospective reflections they could share about their experiences. We did not attain medical records to verify the age of diagnosis. Participants were recruited from the Diabetes Education Resource for Children and Adolescents (DER‐CA), the sole interprofessional paediatric diabetes programme in Manitoba. The DER‐CA offers paediatric specialty services to > 1000 children and youth with diabetes from Manitoba and Northwestern Ontario, of whom > 450 live with T2D. A heterogenous sample was desired; we attained participants largely through convenience sampling using clinic‐based recruitment (e.g., posters, clinicians discussing the study with eligible participants). Interested participants then contacted study coordinators. We had the opportunity for more targeted sampling by telephoning previously consented, eligible participants from the iCARE cohort study (i.e., Improving Renal Complications in Adolescents with Type 2 Diabetes; Dart et al. 2014), in reference to their age and sex. Here, we called participants who had consented to further contact, targeting first those sexes and genders less represented in our emerging sample. In deviation from our protocol, we did not collect anthropometric data given the requirement to transition to virtual interviews due to COVID‐19 research restrictions.

Twenty‐two youth, age 15–25 years, were recruited. Table 1 reports sociodemographic characteristics of participants. Age of diagnosis was not reported; all participants were diagnosed before 18 years of age; many were unable to recount specifically the age of diagnosis.

**TABLE 1:** Socio‐demographic characteristics of study participants (*n* = 22).

Characteristics	*n*
Gender	
Female	13
Male	7
Prefer not to indicate	2
Age	
15–18	10
19–22	9
23–26	3
Location	
Urban	12
Township	4
First Nation	5
Ethnicity	
First‐Nations	15
Hispanic	2
White	2
Metis	1
Afro‐Caribbean	1
Asian	1

### 2.4 Data Collection

Data were collected between February and March 2021. A semi‐structured interview guide was developed in response to existing qualitative research on youth onset T2D which demonstrated a need for exploratory questioning of experience and perceptions and was discussed extensively within the team. Interview questions were pilot tested, proceeded from the general to the specific, and centred upon youths' experiences of T2D.

Twenty‐two semi‐structured interviews were conducted. Interviews ranged from 19:11 min to 53:56 (Mean: 29:01). Interviews were conducted virtually using telephone (*n* = 19) or Zoom audio (*n* = 3) at the request of participants; only the participant and researcher were present throughout data collection. All interviews were led by female research assistants with master‐level graduate degrees who received training in qualitative methods, had no previous relationships with study participants, and whose motivation was to provide a safe environment for qualitative data collection. Participants were informed about the study purpose and encouraged to share their perspectives openly.

Following a short icebreaker to facilitate rapport, the interview commenced with the question: I understand you have T2D; can you tell me about what this means to you? Examples of other questions included: What is most important to you in terms of living with diabetes? What would you like health providers to know about your experience of living with T2D? Open‐ended follow‐up questions were also used, such as ‘can you tell me more about that?’ All interviews were audio‐recorded. Field notes and analytic memos were taken during and after the interviews to contextualise data and facilitate analysis. Data collection continued until data saturation was attained—as assessed during the interviews—and later confirmed by code saturation (Rahimi and Khatooni 2024). All interviews were conducted over the telephone or using Zoom video conferencing, previously shown to be a reliable and desirable interview method with comparable data quality (Archibald et al. 2019).

### 2.5 Data Analysis

Interviews were recorded, professionally transcribed verbatim, cleaned, and uploaded to MAXQDA 2020 for management (VERBI Software 2019). Transcripts were not returned to participants for comment, and member checking was not undertaken. This aligns with critiques of member checking as misaligned with the thematic levelling of data (i.e., epistemological mismatch) (Thorne 2008). Instead, real‐time validation techniques, such as checking interpretations at the time of data collection through ongoing dialogue, reflexivity, and peer debriefing within the research team through critical review of emerging themes, were conducted throughout the process, aligned with the Interpretive Descriptive approach undertaken.

Inductive thematic analysis followed Braun and Clarke (2006). The first stage, familiarising with the data, involved the lead author (MA) listening to recorded interviews while reading the transcripts to gain a sense of the whole. The second stage involved generating initial codes. Here, the first author conducted line‐by‐line coding from the first five interviews. Coding was open—informed from the data—and descriptive, based on explicit content (e.g., fear as expressed by participants); increasingly interpretive (e.g., low knowledge as determined by participant expression); and accompanied by code‐meaning descriptions and a logbook of emerging analytic reflections. Codes were continually applied to the remaining data set; modified and combined as needed, in constant comparison of the codes to the concepts of experience and perceptions. New codes were recorded in the logbook and applied to previously coded transcripts. Stage three involved the identification of patterns within and across the codes to identify themes and subthemes. This was supported by mind mapping of codes and their relationship, and concurrent examination of code frequency and coverage within and across interviews. An inductive approach was maintained, wherein highly occurring codes were clustered into themes, and clustered codes and emerging themes were considered narratively to avoid categorical representations that appeared non‐supplemental to the research topic (e.g., theme of ‘experiences of T2D’, versus thematic narratives reflecting the nature of this experience). The fourth stage involved a review of the themes. As part of this process, the first author further examined less represented codes for insights into outliers, identified emerging analytic subgroups to aid in comparative analysis, and modified previously identified thematic labels as needed (Thorne 2008; Braun and Clarke 2006). Provisional themes were shared with the research team for critical review; this resulted in two themes being merged and one subtheme discarded to ensure adequate evidential support. In stage five, themes were labelled and their subthemes refined for clarity.

### 2.6 Ethical Consideration

The Education and Nursing Research Ethics Board at the University of Manitoba approved this study (May 2020) and this study received site impact approval (June 2020). All participants were informed about the study and their right to withdraw at any time. Informed consent was attained verbally and was recorded, given the mode of data collection.

### 2.7 Rigour and Reflexivity

Qualitative trustworthiness criteria were used to assure research quality (Tobin and Begley 2004; Lincoln and Guba 1985), such as promoting dependability through analytic memos and note taking; upholding confirmability through the linking of interpretations with data through analytic substantiation; and ensuring methodological congruence of all research components for internal coherence (Thorne 2008; Thorne et al. 2004). Credibility was promoted through the inclusion of a heterogeneous group of participants with varied localities and age ranges of youth. Such diversity increases the likelihood that a range of experiences of T2D are reflected, thereby contributing to a more robust representation. Findings are reported according to the CORE‐Q checklist (Tong et al. 2007) (supplemental file).

## 3 Results

**FIGURE 1:**
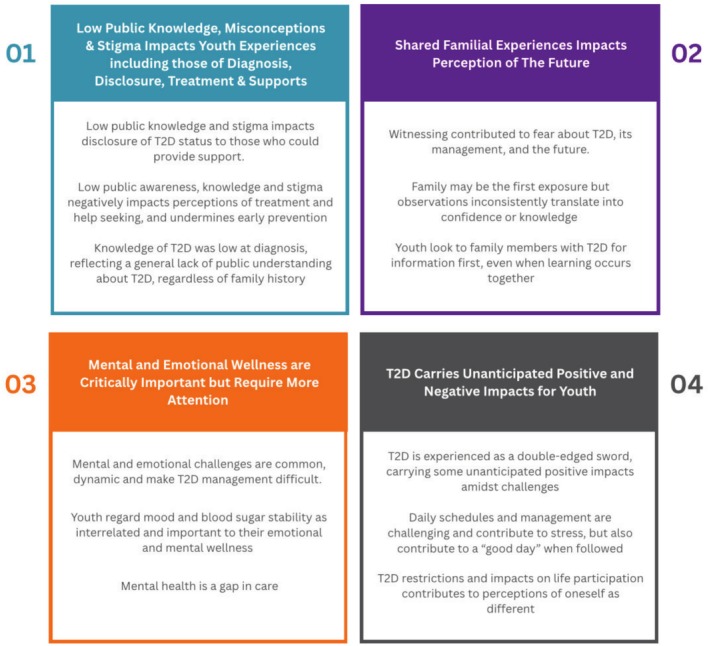
Visual depiction of themes and subthemes. [Colour figure can be viewed at wileyonlinelibrary.com]

The results consist of four themes describing experiences and perceptions of living with T2D. These include: (1) low public knowledge, misconceptions and stigma impact youth experiences including those of diagnosis, disclosure, treatment and supports; (2) shared familial experiences impact perception of the future; (3) mental and emotional wellness is critically important but requires more attention; and (4) T2D carries unanticipated positive and negative impacts for youth (see Figure 1). Throughout the results, participant numbers are indicated beside synthesised statements and quotes to indicate distribution of thematic content across the participant sample.

### 3.1 Low Public Knowledge, Misconceptions and Stigma Impacts Youth Experiences Including Those of Diagnosis, Disclosure, Treatment and Supports

This theme reflects how misconceptions about T2D, coupled with low public knowledge and real or perceived stigma, impact how youth experience diagnosis and T2D management.

#### 3.1.1 Low Public Knowledge and Stigma Impacts Disclosure of T2D Status to Those Who Could Provide Support

Participants discussed how public knowledge around T2D remains low and T2D‐related misconceptions are common. That T2D is associated exclusively with sugar intake and large body sizes was perceived as misconceptions. As one participant indicated, ‘more people who don't have type 2 diabetes should understand that it's not like I ate too much sugar and now I have diabetes. There's more to it than that.’ (P8). This was mirrored by another participant's sentiment that not all people with T2D are visually identifiable as ‘really big, fat people…. they could be like your really skinny neighbour next door’ (P5).

Low public knowledge and misconceptions about T2D appeared connected to public and internalised stigma and impacted whether participants disclosed their T2D status. Participants discussed fear (e.g., of school bullying) if their T2D diagnosis was revealed: you also want to tell your friends and be like, ‘Hey, you know, I'm diabetic’ but. … you know word could get out and you could get teased about it at school (P15). Keeping a T2D diagnosis private protected against anticipated difficulties: ‘Mental health comes into play with it, if your friends – sometimes people will make fun of you’ (P2).

Other times, hesitating to disclose T2D status was related to embarrassment or fear: I don't know why I was so scared to like tell people I had it. I don't know why I was so embarrassed even though it was just like my body and genetics, you know (P22, female). A male participant echoed this statement: ‘At first … I thought diabetes was a bad thing, so I never told nobody about it … But since I was 18, I've just realized to express it – it's not a bad thing, it's just a diagnosis most people get’ (P12). Conversely, T2D awareness provided opportunities for support, ‘Recently … more information is being found out about diabetes and exercise’. So, like some people are like, ‘Oh, you have diabetes. Do you need sugar? Is your blood sugar alright?’ (P14).

During school years, T2D student education was seen as important to reducing associated stigma. There is a need for ‘more education … on diabetes because there's lots of bullying with that’ (P5). Teacher discretion when discussing diabetes was valued, demonstrated by *lowering voices and avoiding public reminders regarding T2D management*. Student education was considered the responsibility of someone else (i.e., an educator), rather than the student with T2D.

Participants regarded teachers as at least partially supportive or at minimum aware and checking in. Participants felt supported when the school nurse was ‘looking out for you’ (P18), or when a teacher ‘would ask me or like if I was feeling sick she would ask me if I need anything’ (P16). Teachers who showed interest in learning about T2D and providing books on T2D were regarded as particularly helpful. Flexibility was an important way of showing support (e.g., flexible snack time, being understanding about absences, food modifications). However, some participants indicated that teachers were not aware of their T2D status, despite at times T2D being indicated on their school registration form. One participant expressed that a teacher didn't take the time to learn about T2D because it didn't personally affect her or teachers didn't care. Some participants voiced they would appreciate more understanding and being looked out for.

#### 3.1.2 Low Public Awareness, Knowledge and Stigma Negatively Impact Perceptions of Treatment and Help Seeking, and Undermine Early Prevention

Participants reflected on the role of stigma, low awareness, and knowledge of T2D. This included public perceptions of T2D and the subsequent impact of these perceptions on T2D management. This was exemplified by one male participant who, when asked if anything makes T2D difficult to manage, emphasised the role of stigma: ‘I do think that the bad rep or stigmatism with diabetes [makes it hard to manage]’ (P14).

At times, participants recognised that low public awareness and knowledge about T2D—including implications of blood sugar fluctuations on behaviour and the use of insulin needles in public—created undesirable circumstances for management. Here, stigmatisation or association of needle use with illicit drug activity was a reflection for one participant: ‘Some public might not like it, because some people might need to take their insulin at certain public places, you know. The wrong imagine in their head, because some might not be …, acknowledged on what a needle is, like, a diabetes needle’ (P12, male). Another participant's stigmatised experience with a patrol police officer during a misinterpreted period of high blood sugar reinforced the need for reduced stigma and public understanding.

Low public awareness and knowledge of T2D impacted how youth perceived their individual risk, and how they approached early lifestyle and prevention efforts. Participants often wished they had known more and would have made different decisions: ‘I wish I could have known, like, how fast it could affect you … I would have tried to make a change’ (P11). Healthy eating and the impact of diet were key interests for earlier education, and should be commonplace, like in schools (e.g., ‘It's not really taught in school’ (P21)), as was knowledge of healthy eating within the family: ‘I wish I knew that both my parents were diabetic; that way I could've like kind of at least like optimistically made better choices as a kid but as a kid when you see Pepsi it's like, “Ooh Pepsi”’ (P15). These centred the locus of control on individual behaviour.

#### 3.1.3 Knowledge of T2D Was Low at Diagnosis, Reflecting a General Lack of Public Understanding About T2D, Regardless of Family History

Even with a family history of T2D, individuals' knowledge of T2D was often low at diagnosis. Most commonly, participants expressed how they did not know anything about T2D at the time of diagnosis. As one participant stated, ‘I think I was like 13 when I got diagnosed, so it was challenging because I didn't know what it was at the time’ (P3).

However, many youth with a family history had some exposure to T2D management or complications, which, in the absence of additional information, helped shape their perceptions about T2D. Negative associations of T2D such as it being ‘dangerous’ (P3), associated with severe complications such as limb loss, or ‘making you very sick’ or ‘get very bad diseases’ (P2, 5), were noted. This general awareness of complications or associated T2D management was present for some youth with previous T2D exposure, either through their communities or by way of family history. This sentiment was exemplified by a male participant who, when asked what he knew about diabetes before diagnosis, responded ‘Not much about it. I was – I mean it runs in my family, I think. … my great‐grandma … She lost her leg … from diabetes.’ (P6). Other participants with family history noticed that insulin was required for management but as a child, ‘didn't really pay attention’ (P9).

### 3.2 Shared Familial Experiences Impacts Perception of the Future

Participants discussed gaining some awareness of T2D through family exposure. Awareness occurred primarily through witnessing rather than family‐based sharing. At times, this contributed to fear about T2D and its management. Despite this, family was discussed as a key source of information and support for participants.

#### 3.2.1 Witnessing Contributed to Fear About T2D, Its Management, and the Future

For most, some aspects of T2D management were visible (e.g., blood glucose, dietary monitoring). However, talking and learning about T2D was also a source of fear: ‘I didn't really know much about it … I was really scared to talk about it.…’ (P11). Observing did not always equate to learning; observing needle use contributed more to fear than to medication knowledge.

Exposure to complications was common for those with a family history of T2D and contributed to fear of the future. Losing limbs was a predominant source of fear, particularly at the time of diagnosis before provider‐based education was initiated. As one participant indicated, ‘It was more of getting told if you get diabetes your limbs will fall off. That was like when I was 12 … (P15). Witnessing the loss of limbs of family members contributed to fear: “my grandparents and my dad, they're diabetic, too. And, like, I have other grandparents that are diabetic. And I've seen them, like, without any – without a leg or something and being sick. And that's just, like, a fear of what if I become like that, too. What if it happens to me early?”’ (P13). Fears from observations also extended towards kidney failure resulting in dialysis, growing a family or caring for existing children, not having their T2D improve, being on life‐long insulin or encountering ‘bad diseases’ (P5). Death was also raised as a fear by one participant.

#### 3.2.2 Family May Be the First Exposure but Observations Inconsistently Translate Into Confidence or Knowledge

As participants witnessed their family members navigate T2D to varying extents, they were provided with numerous opportunities to learn about T2D based on observing affected family members. Understanding that family members had to watch what they eat, monitor their blood sugar, and administer insulin or take medications was common observational learning. Being included in aspects of management helped participants learn and retain information: ‘Grandma always showed me how she injected herself and I always saw grandma and grandpa poke their finger, so I kind of learned that way’ (P18). Observations were infrequently accompanied by knowledge sharing within families, due to ‘*being secretive*’ about T2D (P4), difficulties with personal coping, or a lack of perceived relevance of T2D before a diagnosis was attained. At times, youth indicated that they ‘didn't ask’ (e.g., P10) about T2D despite having a close family member with the diagnosis.

#### 3.2.3 Youth Look to Family Members with T2D for Information First, Even When Learning Occurs Together

When asked where they would seek information, youth often turned to family members such as parents, a grandparent, or an aunt, for example. Google was also a common source of information seeking, and at times was the first method of information seeking. Doctors, specifically those at a specialised clinic for diabetes education and care in the urban centre, were mentioned as key sources of information, particularly for the physical components of disease management. However, they were infrequently the first point of contact for T2D‐related questions, and at times were regarded as ‘less helpful’ than family members who provided support alongside the youth and often learned with them over time.

Learning together was positive for participants, and even those family members with T2D were not expected to have all the answers. Participants described how they ‘google these things together if we didn't fully understand what it was*’* (P9) and doing this together advanced their learning. The positive regard for learning together even extended into friendships, with a youth indicating that they don't speak to friends about T2D but would do so if they had a question, stating that ‘Or better yet … we all learn together’ (P9).

### 3.3 Mental and Emotional Wellness is Critically Important but Requires More Attention

Participants emphasised the importance of mental health in relation to their T2D. This theme extended towards the interrelatedness of mood and blood sugars, mental health as a gap in care, and the need to approach T2D holistically.

#### 3.3.1 Mental and Emotional Challenges Are Common, Dynamic and Make T2D Management Difficult

Youth recognised the importance of the physical aspects of T2D management, such as monitoring A1C, daily blood sugar, and watching their dietary intake. However, many also experienced mental health challenges that impacted their wellness and ability to manage their T2D. T2D was seen to ‘take a toll on you mentally’ (P4). This was captured by one youth who indicated, *‘*another thing that is hard to manage my diabetes is when like with my mental health I have sometimes and then breakdowns or binging episodes and realizing I shouldn't have done that kind of thing and then I feel crappy for myself*…*.’ (P4). Being hard on oneself was present when participants did not follow their management plan. Feelings associated with a bad day or week were seen to directly impact ease of self‐management.

While many participants referred more broadly to mental or emotional challenges, others used more specific words when describing their well‐being. Many participants described experiencing depression related to, and impacting, their T2D. This complicated management, particularly when participants were unable to identify which factors resulted in feeling unwell. As one youth indicated, ‘waking up is kind of a mix of, ‘Is it my blood sugar? Or is it my depression?’ (P14). Less commonly, participants described nervousness, anxiety, or losing hope and not caring what happens with T2D management. Having a supportive team in place and reconnecting with cultural traditions was important to these participants.

The relevance and impact of mental and emotional health on participants in relation to their T2D was not always immediately apparent. Mental and emotional stressors changed overtime, depending on participants developmental stage, roles, and nuances of their treatment and self‐management journey. While challenges with adherence to medications resulted in despair and frequent crying for one participant, more commonly, participants expressed mental health impacts of having or disclosing T2D in schools for fear of being targeted through teasing or bullying, indicating specific stressors pertinent to life stage. Other times the developmental journey illuminated the importance of mental health for participants, and this grew overtime.

#### 3.3.2 Youth Regard Mood and Blood Sugar Stability as Interrelated and Important to Their Emotional and Mental Wellness

While mental and emotional challenges impacted T2D management, T2D itself was seen as impacting mental and emotional wellness. Often, this related to the impacts of blood sugars on mood. Irritability, fatigue, and general moodiness were directly impacted by blood sugar variability: ‘we'll be out and about like for hours sometimes I am able to keep up with them, but sometimes my mood drastically changes because the physical, just physiological stuff that I'm feeling. Like very tired now and irritable because it's either like I have low sugars or too high, so it's a direct effect for sure of having type 2 diabetes or having to manage it every day’ (P1). This was mirrored by many participants who experienced the mental and emotional impacts of blood sugar spikes and dips, often in the form of mood swings.

Managing blood sugars was challenging for many participants. The volume and rapidity of blood sugar changes were a grievance and impacted how well participants felt throughout the day. As one participant indicated, ‘I woke up with a low blood sugar and I felt crappy. …it all depends on what that blood, what that number is’ (P3). Rapid changes in blood sugar impacted mood and influenced whether it was a good day. Frequently, a good day with T2D was when a routine could be completed without moodiness: ‘… I would also take my medication and I would come home and I wouldn't be moody and so like, I'd have a good day’ (P21).

The link between blood sugars, mood, and mental wellness was highlighted to stress the importance of management. Blood sugar was a key component of T2D management because of its impact on mood and mental health. Having blood sugars under control meant that participants could focus on other aspects of their wellness and quality of life and avoid the impact of high blood sugar on ‘mood being all over the place’ (P5). As one individual indicated, ‘when I keep my blood sugar level normal I have—I can like focus on quality of life and keeping me—doing happy stuff and fun stuff.’ (P8).

#### 3.3.3 T2D Should Be Viewed Holistically Given the Mental Health Impacts Experienced by Youth

Individuals frequently identified that T2D was more than just the physical components; ‘it's more of a physical and emotional’ (P21). Mental health was seen as integral to or most important, a ‘*big part*’ (P4) of T2D, or on equal footing with physical, emotional, and spiritual wellness. One participant exemplified the need to view T2D holistically when stating, ‘diabetes is linked to everything, even your mental health’ (P4). This was frequently related to blood sugar and at times stigma (theme 1). Additional impacts of T2D on participants are discussed in theme 4.

Over time, participants recognised life stressors, needed supports, and their impact on T2D management. Mental health and its role in T2D became apparent, and many participants appreciated the interconnectedness of their mental, emotional, and physical health. One participant described how the busyness of work impacted blood sugars when he got home: ‘Aw. Thank God I'm home. I can actually take a break… I find that stress, stress, stress makes sugars go up’ (P6). While holistic wellness was often mentioned, spiritual health was explicitly identified as important to participant 8 and was seen as contingent upon mental wellness: ‘if you don't take care of your mental health then you can't take care of your physical or spiritual health.’

#### 3.3.4 Mental Health Is a Gap in Care

Recognising the importance of mental and emotional health in T2D, participants often expressed how more support is needed. Participants noted a lack of mental health discussions with providers or desired more; mental health was uncommonly discussed in the context of the healthcare provider–patient relationship. Gaps in mental health care in these encounters often manifested as a lack of checking in, or asking how a participant was doing mentally or emotionally. As one participant expressed, ‘no one really touched base on how I was actually doing mentally about the whole thing’ (P1).

Participants were generally positive when discussing their relationships with their T2D‐specific health providers in terms of kindness and trust. However, they also recognised that a lack of attention to mental and emotional health impacted the approach to treatment. Sentiments of ‘seems they just want to solve everything with medication’ (P8), ‘focusing more on dieting and A1C’ (P4), and ‘quite a bit of lack of focus on the holistic part’ (P1) were common. Participants wanted healthcare providers to know that their experience of T2D went beyond the ‘heavy toll’ of the physical … and took a really heavy toll mentally’ (P11). Recognising that the mental and emotional components of T2D were integral to their experiences was seen as important to improving care.

Mental health responsibility in treatment was unclear and diffuse. Many participants desired mental health to be more central in the healthcare‐patient interaction space, while others questioned whether supporting mental health was the job of the T2D clinic healthcare providers. Some took responsibility for learning about mental health impacts independently because the clinic staff ‘don't really talk about that kind of stuff’ (P20). Other times, a lack of integrated mental health support resulted in family members advocating for or mobilising mental health resources on behalf of the participant. As one participant indicated ‘Well that one [mental health] they didn't exactly tell me. My mom kind of like asked them to get me a therapist because I was dealing with like depression at the time. Yeah, so my social worker she taught me some skills like breathing and she talked me through it, yeah’ (P5). Providing access to a therapist was identified by other participants to improve integration of mental health into service delivery.

### 3.4 T2D Carries Unanticipated Positive and Negative Impacts for Youth

Youth expressed several impacts related to T2D. Some impacts were negative, such as limited participation in social encounters. However, some impacts were positive and unexpected. Routines and schedules were identified as key to improving daily wellness and reducing the negative impacts of T2D, although the impacts of T2D on perceptions of the self as different persisted for many participants.

#### 3.4.1 T2D Is Experienced as a Double‐Edged Sword, Carrying Some Unanticipated Positive Impacts Amidst Challenges

Many participants expressed that despite the challenges and stresses experienced with T2D, there were positives associated with their diagnosis. For some, T2D prompted reflection on past choices and recognition of positive lifestyle changes. As such, positives often related to healthy lifestyle changes, and predominantly, healthy eating and exercise. As one participant indicated, ‘it's been a challenge for me because there's complications popping up now since I didn't really take good care of myself now that I realized … I used to think of it as like the negative part of my life but at the same time it's very positive … I make better choices for my body and other stuff that doesn't – but the negative has probably, my complications, and making sure I'm aware of like every day my blood sugars and all that*’* (P4). A mix of positive and negative components was pervasive in the sample, and captured in the statement, ‘In a way, it kind of saves your life, but doesn't, I guess, you know, like, in a non‐healthy way, I guess’ (P9). A feeling of being pressured into rapidly making changes was often difficult for participants.

While improvements to diet and activity level were the most common positive changes identified by participants, awareness of health was another frequently cited positive. Here, participants gained awareness of the need to take care of themselves, their health and their bodies; and became more in tune with their bodies. At times, this awareness and associated lifestyle changes were ‘eye opening’ for participants. While a sense of accomplishment or empowerment was observed during the interviews, one participant explicitly identified that these changes contributed to a sense of empowerment.

Relationships were another area identified as including both positive and negative components related to T2D and its management. At times, the nature of friendships improved and was contingent upon whether T2D was disclosed to friends. Participants described friends or family becoming more supportive, more inclusive, and some relationships becoming closer, which they attributed to having T2D. However, others expressed feeling like a burden for the required lifestyle changes and sickness associated with T2D. Two participants expressed no positives related to T2D.

#### 3.4.2 Daily Schedules and Management Are Challenging and Contribute to Stress, but Also Contribute to a ‘Good Day’ When Followed

Participants found daily management and maintaining a routine among the most challenging and impactful aspects of T2D. As one participant expressed, ‘it's very impactful, especially even just doing daily stuff. Sometimes if you forget to take the dose or you end up eating too much of something that is not usually recommended for you to have type 2 diabetes, it can really affect like your life’ (P1). Slight changes in schedule created undesirable impacts, such as increases in blood sugar, or forgetting or delaying medication and consequently feeling unwell. As such, for many participants, a good day with T2D was described according to staying on routine or schedule.

Different strategies for maintaining schedules or routines were identified. However, many times, participants referred generically to the need to maintain routines but did not identify specific methods that helped them accomplish this goal. Familial support, such as reminders, was helpful to some participants, while others benefited from strategies such as phone or note reminders. Routinising or becoming comfortable with the schedule improved over time for some participants as they gained confidence and independence with daily management.

The constant vigilance required of participants was highly challenging, particularly as it related to dietary intake, medication management, and blood sugar monitoring. This was frequently described as stressful; and forgetting to take medication or test blood sugar caused difficulties. As one participant stated, ‘I'm kind of bad at remembering to take my meds all the time. Or like remembering to test my blood sugar. And like sometimes it's hard to eat healthy and then my blood sugar gets higher. And that stresses me out.*’* (P8). Lack of motivation or maintaining motivation as it relates to exercise, diet, medication, or blood sugar monitoring was a commonly identified barrier to maintaining such vigilance.

#### 3.4.3 T2D Restrictions and Impacts on Life Participation Contributes to Perceptions of Oneself as Different

Participants commonly described that T2D changed the extent and nature of participation in their lives. They described experiencing more restrictions–predominantly regarding food, drinks and exercise. At times, restrictions were seen as a part of life: ‘I'm able to live like a basically normal life but with a little bit restriction here and there’ (P5). However, others expressed more pronounced restriction through statements such as, ‘I can't go anywhere far from my home … I can't do anything; I always have to monitor it’ (P17).

For some, T2D management felt like a barrier to participation in daily activities. These participants expressed feeling limited and unable to engage how they wanted to. One participant felt ‘limited to what I can do and I feel like if I try to learn something … I won't be able to do it to the best of my ability’ (P11). Participation in sport and modifying the nature of social participation with friends or family were poignant examples of participants' experiences of limitations.

T2D‐related restrictions often sparked a desire to fit in (e.g., eat like others) or compare life before diagnosis. These changes contributed to an undesirable perception of the self as different. Participants described how aspects of their T2D management made them feel ‘not normal’ compared to others: ‘I felt like not normal with other people. Because I couldn't do – like eat what they could eat as much as they could eat and I had to always do insulin, which was a problem’ (P2). Participants disliked activities that drew attention to them, as these intensified feelings of difference and isolation. For some, a sense of normalcy was regained over time, as familiarity with the management routine was gained and supports were activated. This same participant captured this sentiment well: ‘I pretty much feel normal and like after I got it under control. But after a while you get used to it, it feels normal but at the start it doesn't feel normal. Not one day you feel like you're just not a part of this world, like you're left out, you're the only one’ (P2).

## 4 Discussion

This qualitative inquiry revealed a complex interplay between external factors, such as public perceptions of T2D, misconceptions and stigma; exposures and shared family experiences which shape fears and expectations around T2D; and participants' experiences and perceptions of T2D. It highlighted the need for a holistic approach to youth onset T2D inclusive of mental and emotional wellness in the T2D journey. Unanticipated were the positive components of T2D and its management, which resulted in stronger relationships and healthy decision‐making in the lives of many youths in our sample. This work complements and extends the findings of the small number of qualitative research studies in youth onset T2D, noting that these studies focus on life transitions (Rasmussen et al. 2016), utilise a small number of research participants (e.g., *N* = 8; Protudjer et al. 2014; Salamon et al. 2012), or specifically explore social support (Brouwer et al. 2012; Huynh 2016) or self‐management (Huynh 2018; Mulvaney et al. 2008). A larger mixed sample size from a recent investigation in Western Australia included 8 adolescent participants from the total sample of 24 and contributed a similar framing of shame and diagnostic concealment (Carman et al. 2023). Further, a recent mixed focus group investigation (i.e., youth, parent, health care provider participants) to inform a mind–body intervention in youth with T2D further highlighted the dearth of qualitative evidence in this area and emphasises the impact of psychosocial and emotional factors on self‐management (Bransteter et al. 2024). This in‐depth qualitative inquiry exclusive to youth experiences and perceptions of T2D adds significant perspective to this literature.

For instance, in a recent investigation, Wicklow et al. (2021) approached the perspectives of Indigenous youth living with diabetes using interpretive description. They identified the trauma of diagnosis, fear of complications, understanding the cause of diabetes, self‐surveillance, and support as five themes central to these perceptions. Like theme 1, which positioned a need for increased understanding, reduced misconceptions and stigma to support a positive T2D experience, a desire for increased understanding of T2D from peers, family members, and health care providers was identified. This finding was further extended by noting that the endemic nature of T2D in Indigenous communities resulted in many youth ‘bearing witness’ to familial T2D, which modified the level of knowledge held about T2D at the time of diagnosis, making this more traumatic for youth. As a unique contribution, youth participants in this study expressed knowledge and fear around T2D‐related complications (Wicklow et al. 2021). This finding was both supported and extended by themes 1 and 2 in our current study, which highlighted unchallenged assumptions and misconceptions about T2D, including what can be done to prevent complications and the possibility of living well with T2D despite poor family outcomes. Witnessing was often a source of fear, underscoring the importance of approaching T2D as a family illness with knowledge, with intergenerational considerations and impacts, including the possible benefits of knowledge sharing between family members.

Aligned with stigmatising public misconceptions, including that T2D results from poor individual choices including laziness and high sugar intake, participants often expressed how earlier knowledge of personal risk factors for T2D—including knowledge of family history—may have encouraged different choices (themes 1 and 2). However, although diet and physical activity reflect a portion of the equation in disease development and progression, an overemphasis on individual choices negates the more systemic factors that overwhelmingly contribute to the growing rates of T2D in youth (McGavock et al. 2017). Factors such as food insecurity, poverty, and lack of health service access are underrepresented as risk factors in the literature yet profoundly contribute to the disproportionate number of Indigenous youth with youth onset T2D. Displacement from the land, disconnection with traditional food sources, and familial disruptions secondary to colonial policies (e.g., residential schools, the 60s scoop) are critical contextual factors contributing to the growth of youth onset T2D and should be considered as such (McGavock et al. 2017). Public awareness and educational initiatives aimed at targeting misconceptions and stigma are necessary to provide the broader systemic context within which T2D develops and is managed. School‐based educational initiatives targeting early learning would be beneficial given the infrequent supports youth experienced at schools in relation to their T2D, including protecting privacy, offering accommodations, and encouraging participation in school‐based activities. Public awareness of T2D may help reduce assumptions around self‐management behaviours (e.g., needle use in public), thereby supporting confidence and reducing anxiety around how self‐management is publicly perceived.

The high emphasis on individual choice may also reflect an internalised stigma or self‐blame within our sample. Participants often expressed how they wished they had known more and may have made different lifestyle decisions (e.g., *I would have tried to make a change*; P11). Within the sample, healthy eating and the impact of diet was a key interest for earlier education, and participants felt this should be commonplace in places like in schools. The reflection on what could have been known, where they could have learned, and what was missed points to the range of spaces and places where such education could be delivered, and that these respective institutions or organisations may not fully be achieving their mandate to support healthy youth development, rather than youth internalising and blaming themselves as though it is exclusively an individual risk.

Fear was another common experience reflected in our findings. Fear was often expressed in relation to self‐management (e.g., needle‐based medications) and complications. While the nature of this fear was not the focus of this inquiry, analysis indicated that family exposures and impacts on self‐management were two dimensions worthy of attention and that fear may be important to address during health care encounters and education. Regarding self‐management, participants expressed not wanting to talk about T2D due to fear about the disease and possible complications or disclose T2D status due to fear of social repercussions (e.g., bullying). As such, fear has behavioural implications since disclosing T2D status to trusted peers and family was an important source of support for youth able to do so in our sample. Fear may infiltrate other aspects of self‐management beyond disclosure or discussion, such as avoiding blood sugar checks due to not wanting to know the results.

As explicated in theme 2, fear related to complications was intimately connected to family exposures around T2D, wherein complications were observed. Families in our sample were unlikely to discuss T2D with the youth participant early on; discussion of the likelihood of complications and strategies to live well to avoid these complications occurred infrequently. Education needs to incorporate the family experience and the likelihood of youth being exposed to T2D early on, and other intergenerational aspects of T2D such as shared risk factors. Engagement with community co‐researchers has suggested that there may be protective reasons why such discussions may not take place; and even with support within families, it cannot be assumed family members with T2D will proactively share experiences in the form of preventative education for youth. It can be understood, however, that youth with T2D often seek and value information and support from family members, highlighting family as an important communication channel for T2D information and education.

A key and actionable finding from this research (theme 3) is that, while physical health is certainly important to youth, their mental health challenges are pervasive and often overlooked. Awareness that many individuals experience emotional and mental challenges secondary to blood sugar fluctuation is important, not only for self‐awareness and management, but also as a pre‐emptive aspect of education within the clinical encounter. There is a resulting need for more robust and consistent mental health research and support in this space, including a developmental perspective on mental health support, given the mean age of diagnosis, and because stressors, experiences and development change over time.

## 5 Strengths and Limitations

This study sampled participants from a province that has the highest rates of youth onset T2D in Canada. Based on the breadth of our sample and thick description, we expect some transferability of findings but acknowledge a larger geographic sample may reveal differences secondary to sociodemographic characteristics and health resource provision. We relied on self‐report of T2D diagnostic age, which was inconsistently recalled and reported. Limited patient and public involvement was incorporated in the design of the study. Extensive involvement came after study conclusion, focusing on the co‐development of KT tools reflecting the study findings.

## 6 Recommendations for Further Research

Findings from this research shed light on the mental health challenges and social complexities involved with youth onset T2D and highlight areas where myth dispelling through public health messaging and initiatives would be helpful. A holistic and youth‐centred approach that provides anticipatory support is therefore warranted. Locally, for example, clinical team involvement in this and adjacent research has resulted in a local practice change (i.e., a template to routinely inquire about mental health within a diabetes clinic). The frequent mention of routines or schedules to achieve a ‘good day’ with T2D is another area where practical supports could be useful and subsequently examined within a co‐design and evaluative research framework. It is unknown what tools would help with the implementation of schedules, or which schedule features would be most beneficial.

Meaningful life participation was an important aim for participants in this study. Participating in sport and needing to modify the nature of social participation were poignant examples of the limitations experienced (future research could explore with participants which tools, if any, could support safe and fulsome involvement in these activities and explore safe modalities for communicating T2D across the various contexts in which youth participate, e.g., school, community, sport, workplace). Similarly, there appeared to be a desire for local education and support outside of the medical model of delivery, such as support and knowledge sharing through schools and within the family unit.

There is much to be done in aligning public perception with research evidence on the systemic and structural risk factors impacting youth onset T2D. However, we are commencing further investigation using an interactive virtual platform (i.e., Brightspark Care Lab; Archibald et al. Unpublished manuscript) to longitudinally investigate the mental health experiences of individuals with youth onset T2D– an underexplored domain highlighted within the current study. Recognising the need for KT resources in this space, we are also co‐designing creative KT resources to mobilise findings and target T2D misconceptions.

## 7 Implications for Policy and Practice

The centrality of mental health to overall wellness and the contingencies between mental health and self‐management in youth onset T2D has implications for service providers and health systems. Routinely investigating mental health during clinical encounters, providing anticipatory guidance, and awareness of mental health supports are imperative to a holistic approach to care reflective of the lived experiences of youth with T2D. Such care should reflect attention to ongoing colonial practices, harmful policies, and biomedical dominance that have and can displace Indigenous ways of knowing and healing, thereby impacting both the access to and the experience of mental health service provision (Josewski 2023). Integrating mental health service providers within or adjacent to T2D clinics would offer more accessible supports to youth navigating a complex diagnosis and is consistent with clinical practice guidelines (Shah et al. 2024). Given the distribution of youth with T2D across a large geographic area, there is a concomitant need for further resource availability across the province.

## 8 Conclusion

This study contributed an in‐depth understanding of the perceptions and experiences of individuals with youth onset T2D to inform KT initiatives and clinical care. That mental health concerns were prevalent yet underattended highlights a critical aspect of holistic care that can be further supported. Pervasive misconceptions about T2D and associated stigmatisation—and often internalised stigma—were notable and reflect opportunities to target public and professional knowledge and attitudes through education and disruptive communication strategies, such as creative and arts‐based KT. Further consideration of youth‐centered supports, including encouraging knowledge sharing within families and schools, for example, and those expressed as helpful to adaptation, routine, or ‘living well with T2D’ are warranted, along with continued attention to holistic research and care for youth navigating the complexities of T2D.

## Author Contributions

M.A., A.D., B.W., E.S. – made substantial contributions to conception and design, or acquisition of data, or analysis and interpretation of data. M.A., A.D., B.W., S.M., K.P., E.S. – involved in drafting or revising the manuscript for important intellectual content. M.A., A.D., B.W., S.M., K.P., E.S. – gave final approval of the version to be published. M.A., A.D., B.W., S.M., K.P., E.S. – agreed to be accountable for all aspects of the work in ensuring that questions related to the accuracy or integrity of any part of the work are appropriately investigated and resolved. M.A. is the guarantor of this work and, as such, had full access to all the data in the study and takes responsibility for the integrity of the data and the accuracy of the data analysis.

## Conflicts of Interest

The authors declare no conflicts of interest.

## Supporting information


**Data S1:** jan70230‐sup‐0001‐DataS1.docx.

## Data Availability

The datasets generated during and/or analysed during the current study are not publicly available due to the nature of the data and lack of participant consent to sharing.
