# Supplementary material for: Beyond AIC: An Interpretive Descriptive Qualitative Study of Youth Experiences and Perceptions of Living With Type 2 Diabetes
Source: J Adv Nurs. 2025 Sep 25;82(6):6373–85. doi: 10.1111/jan.70230 (PMC13176690; doi:10.1111/jan.70230)
Supplement: Supplementary file 1 — Data S1: jan70230‐sup‐0001‐DataS1.docx. [file JAN-82-6373-s001.docx]

**Supplementary file**

**Consolidated criteria for reporting qualitative studies (COREQ): 32-item checklist**

**Guidelines for Reporting Qualitative Research**

**Consolidated criteria for reporting qualitative studies (COREQ): 32-item checklist**

| **Item** | **Completed** |
| --- | --- |
| **Domain 1: RESEARCH TEAM & REFLEXIVITY** |  |
| **Personal Characteristics**  **Interviewer/facilitator**  1.Which author/s conducted the interview or focus group? | **Under results**  **“**Interviews were led by the research assistants with master level graduate degrees who trained in qualitative methods |
| **Credentials**  2. What were the researcher’s credentials? E.g. PhD, MD | **Under results**  Masters level graduate training |
| **Occupation**  3.What was their occupation at the time of the study? | **Under results**  Research assistant |
| **Gender**  4.Was the researcher male or female? | **Under results**  **“**female”  ********please note that the researchers sex and gender identity were both female* |
| **Experience and training**  5.What experience or training did the researcher have? | **Under results**  “received training in qualitative methods” |
| **Relationship with participants**  6. Relationship established Was a relationship established prior to study commencement? | **Under results**  “No existing relationship with study participants” |
| **Participant knowledge of the interviewer**  7. What did the participants know about the researcher? e.g. personal goals, reasons for doing theresearch | **Under results**  Purpose of the research was shared |
| **Interviewer characteristics**  8. What characteristics were reported about the interviewer/facilitator? e.g. Bias, assumptions,  reasons and interests in the research topic | **Under results**  Motivated to create a safe environment for qualitative data collection |
| **Domain 2: STUDY DESIGN** |  |
| **Theoretical framework: Methodological orientation and Theory**  9.What methodological orientation was stated to underpin the study? e.g. grounded theory,  discourse analysis, ethnography, phenomenology, content analysis | **Under design**  **“**This is an exploratory qualitative study guided by Interpretive Description (Thorne, 2008). The methods are described in detail in our published protocol” |
| **Participant selection**  **Sampling**  10. How were participants selected? e.g. purposive, convenience, consecutive, snowball | **Under setting and sample**  **“**A heterogenous sample was desired; we attained participants largely through convenience sampling using multiple methods (e.g., clinic-based recruitment), with opportunity for more targeted sampling made possible by contacting previously consented participants from adjacent research, with reference to their age and sex demographics.” |
| **Method of approach**  11.How were participants approached? e.g. face-to-face, telephone, mail, email | **Under setting and sample**  **“**who consented to being contacted about other research were contacted by telephone; … contacting previously consented participants from adjacent research over the telephone” |
| **Sample size**  12.How many participants were in the study? | **Under results**  22 participants |
| **Non-participation**  13**.** How many people refused to participate or dropped out? Reasons? | **Under results**  An additional participant consented to the study, had to leave the interview at commencement, and did not reschedule; no reason was attained |
| **Setting**  Setting of data collection  14. Where was the data collected? e.g. home, clinic, workplace | **Under results**  **“**Interviews were conducted virtually using telephone (n=19) or Zoom audio (n=3) at the request of participants.” |
| **Presence of non-participants**  15. Was anyone else present besides the participants and researchers? | **Under results**  **“**only the participant and researcher were present throughout data collection” |
| **Description of sample**  16.What are the important characteristics of the sample? e.g. demographic data, date | **Under results**  Extensive description provided |
| **Data collection**  **Interview guide**  17.Were questions, prompts, guides provided by the authors? Was it pilot tested? | **Under data collection and analysis**  Pilot tested and general description of the questions; interview guide not provided |
| **Repeat interviews**  18.Were repeat interviews carried out? If yes, how many? | **Under results**  22 individuals completed 22 interviews |
| **Audio/visual recording**  19.Did the research use audio or visual recording to collect the data? | **Under data collection and analysis**  Interviews were recorded |
| **Field notes**  20. Were field notes made during and/or after the interview or focus group? | **Under data collection and analysis**  Field notes and analytic memos were taken during and after the interviews |
| **Duration**  21.What was the duration of the interviews or focus group? | **Under results**  Interviews ranged from 19:11 min to 53:56 (*Mean:* 29:01). |
| **Data saturation**  22.Was data saturation discussed? | **Under results**  Data collection continued until data saturation was attained, as assessed during the interviews, which was later confirmed by code saturation |
| **Transcripts returned**  23. Were transcripts returned to participants for comment and/or correction? | **Under results**  Transcripts were not returned to participants |
| **Domain 3: ANALYSIS & FINDINGS** |  |
| **Data analysis**  **Number of data coders**  24.How many data coders coded the data? | **Under data collection and analysis**  One |
| **Description of the coding tree**  25. Did authors provide a description of the coding tree? | **Under data collection and analysis**  Brief description provided |
| **Derivation of themes**  26.Were themes identified in advance or derived from the data? | **Under data collection and analysis**  **“**clustered highly occurring codes into themes, examined less represented codes for insights into outliers, identified emerging analytic subgroups to aid in comparative analysis, and modified previously identified thematic labels as needed” |
| **Software**  **27.**What software, if applicable, was used to manage the data? | **Under data collection and analysis**  MAXQDA 2020 |
| **Participant checking**  28. Did participants provide feedback on the findings? | Participants did not provide feedback on the findings as per protocol |
| **Reporting**  Quotations presented  29. Were participant quotations presented to illustrate the themes / findings? Was each  quotation identified? e.g. participant number | **Under Results**  Extensive quotations provided, with participant ID numbers |
| **Data and findings consistent**  30.Was there consistency between the data presented and the findings? | Consistent |
| **Clarity of major themes**  31.Were major themes clearly presented in the findings? | **Under Results**  Clearly presented across 4 themes |
| **Clarity of minor themes**  32. Is there a description of diverse cases or discussion of minor themes? | **Under Results**  Sub themes clearly labelled and identified |

(Tong et al., 2007)
